# Association Between Prediabetes and Erectile Dysfunction: A Meta-Analysis

**DOI:** 10.3389/fendo.2021.733434

**Published:** 2022-01-10

**Authors:** Mingyu Jin, Shaoying Yuan, Bo Wang, Luqi Yi, Chenxia Wang

**Affiliations:** ^1^ Department of Andrology, Guangdong Hospital of Traditional Chinese Medicine, Zhuhai, China; ^2^ Department of Infertility and Sexual Medicine, The Third Affiliated Hospital of Sun Yat-Sen University, Guangzhou, China; ^3^ Department of Urology, Guangdong Hospital of Traditional Chinese Medicine, Zhuhai, China; ^4^ Department of Gynecology, Guangdong Hospital of Traditional Chinese Medicine, Zhuhai, China

**Keywords:** prediabetes, erectile dysfunction, prevalence, observational studies, meta-analysis

## Abstract

**Background:**

Diabetes has been associated with the increased risk of erectile dysfunction (ED). However, previous studies evaluating the association between prediabetes and ED showed inconsistent results. We performed a meta-analysis of observational studies to systematically evaluate the above association.

**Methods:**

Relevant observational studies were retrieved by search of PubMed, Embase, and Web of Science databases. A random-effect model which incorporated the potential intra-study heterogeneity was used for the meta-analysis. Subgroup analyses were performed to evaluate the influences of study characteristics on the outcome.

**Results:**

Nine studies (five matched case-control studies and four cross-sectional studies) were included. Age were adjusted or matched in all of the studies. Pooled results showed that compared to men with normoglycemia, men with prediabetes were associated with higher prevalence of ED (odds ratio = 1.62, 95% confidence interval: 1.28 to 2.07, P < 0.001; I^2^ = 78%). Subgroup analyses showed that the association was not significantly affected by definition of prediabetes, diagnostic tool for ED, or controlling of additional variables besides age (both P for subgroup difference > 0.05). However, the association between prediabetes and ED seemed to be stronger in case-control studies than that in cross-sectional studies, and in studies with younger men (mean age < 50 years) than in those with older men (mean age ≥ 50 years; both P for subgroup difference < 0.05).

**Conclusions:**

Prediabetes is associated with higher prevalence of ED, which may be independent of age of the males and may be stronger in young men.

## Introduction

Erectile dysfunction (ED) is a common sexual dysfunction in men, which is defined as the failure to achieve or maintain an erection for satisfactory sexual intercourse (1, 2). The incidence of ED is age-related, and more than 20% of men aged over 40 years were reported to have ED (3). Besides aging, diabetes mellitus (DM) has also been recognized as a major risk factor for ED (4). A previous meta-analysis including 145 studies showed that the overall prevalence of ED in men with DM was 52.5% (5). Accordingly, screening and prevention of ED in men with DM has gained great attention (6).

In recent decades, the intensive care for patients with hyperglycemia has introduced the concept of prediabetes. Currently, prediabetes is defined as an intermediate metabolic state between normoglycemia and DM, which includes impaired fasting glucose (IFG), impaired glucose tolerance (IGT), and mildly raised glycated hemoglobin (HbA1c) (7, 8). Although IGT is consistently defined as a 2 hour plasma glucose concentration of 7.8-11.0 mmol/L during an oral glucose tolerance test, the definitions of IFG are varying according to the World Health Organization (WHO) criteria (fasting plasma glucose [FPG]: 6.1 to 6.9 mmol/L) and the 2003 American Diabetes Association (ADA) guideline criteria (FPG: 5.6-6.9 mmol/L) (9). Moreover, the glycosylated hemoglobin (HbA1c) of 5.7-6.4% and 6.0-6.4% has also been considered as definitions for prediabetes by ADA and National Institute for Health and Care Excellence (NICE) respectively (10, 11). Similar to DM, prediabetes is suggested to be associated with increased risks of cardiovascular diseases, over cancer events, and all-cause mortality (12, 13). However, previous studies evaluating the association between prediabetes and ED showed inconsistent results (14–22). Some studies suggested that prediabetes was associated with higher prevalence of ED as compared to normoglycemia (15, 16, 19, 20, 22), while others did not (14, 17, 18, 21). In this study, we performed a meta-analysis of cohort studies to systematically evaluate the association between prediabetes and ED. The potential influence of different definitions of prediabetes on the association was also explored.

## Methods

The meta-analysis was performed in accordance with the MOOSE (Meta-analysis of Observational Studies in Epidemiology) (23) and Cochrane’s Handbook (24) guidelines.

### Literature Search

Studies were identified *via* systematic search of electronic databases of PubMed, Embase, and Web of Science databases *via* the following terms: (1) “prediabetes” OR “pre-diabetes” OR “prediabetic state” OR “borderline diabetes” OR “impaired fasting glucose” OR “impaired glucose tolerance” OR “IFG” OR “IGT”; and (2) “erectile dysfunction” OR “erectile function” OR “sexual dysfunction” OR “sexual function” OR “ED”. The search was limited to human studies published in English or Chinese. The reference lists of related original and review articles were also analyzed using a manual approach. The final literature search was performed on May 5, 2021.

### Study Selection

The inclusion criteria for the studies were: (1) observational studies published as full-length articles; (2) included adult male participants; (3) evaluated the association between prediabetes and ED; and (4) reported the relative risk for this association as compared men with normoglycemia after adjustment or control of potential confounding factors, at least for age. The definition of prediabetes was based on the criteria of the original articles, and the diagnostic criteria for ED were consistent with those of the original studies. Reviews, editorials, preclinical studies, and studies irrelevant to the aim of current meta-analysis were excluded.

### Data Extracting and Quality Evaluation

Literature search, data extraction, and quality assessment of the included studies were performed independently by two authors according to the predefined inclusion criteria. Discrepancies were resolved by discussion with the corresponding author. The extracted data included: (1) name of first author, publication year, and country where the study was performed; (2) study design characteristics; (3) participant characteristics, including health status, sample size, and mean age; (4) definition for prediabetes and numbers of men with prediabetes; (5) diagnostic methods for ED and numbers of men with ED; and (6) confounding factors adjusted or controlled when the association was reported. The quality of each study was evaluated using the Newcastle-Ottawa Scale (25) which ranges from 1 to 9 stars and judges each study regarding three aspects: selection of the study groups; the comparability of the groups; and the ascertainment of the outcome of interest.

### Statistical Analyses

We used odds ratios (ORs) and their corresponding 95% confidence intervals (CIs) as the general measure for the association between prediabetes and ED as compared with men with normoglycemia. Data of ORs and their corresponding stand errors (SEs) were calculated from 95% CIs or P values, and were logarithmically transformed to stabilize variance and normalized the distribution (24). The Cochrane’s Q test and estimation of I^2^ statistic were used to evaluate the heterogeneity among the include cohort studies (26). A significant heterogeneity was considered if I^2^ > 50%. We used a random-effect model to synthesize the OR data because this model is considered as a more generalized method which incorporates the potential heterogeneity among the included studies (24). Sensitivity analyses, by omitting one individual study at a time, were performed to test the robustness of the results (27). Predefined subgroup analyses were performed to evaluate the influences of study characteristics on the outcome, including definition of prediabetes, study design, mean age of the male participants, methods for diagnosis of ED, and whether confounding factors besides age were controlled. Medians of continuous variables were used as the cutoff values for grouping. The potential publication bias was assessed by visual inspection of the symmetry of the funnel plots. Additionally, the Egger’s regression asymmetry test was further performed for the evaluation of potential publication bias (28). We used the RevMan (Version 5.1; Cochrane Collaboration, Oxford, UK) and STATA software for the meta-analysis and statistics.

## Results

### Literature Search

**Figure 1:**
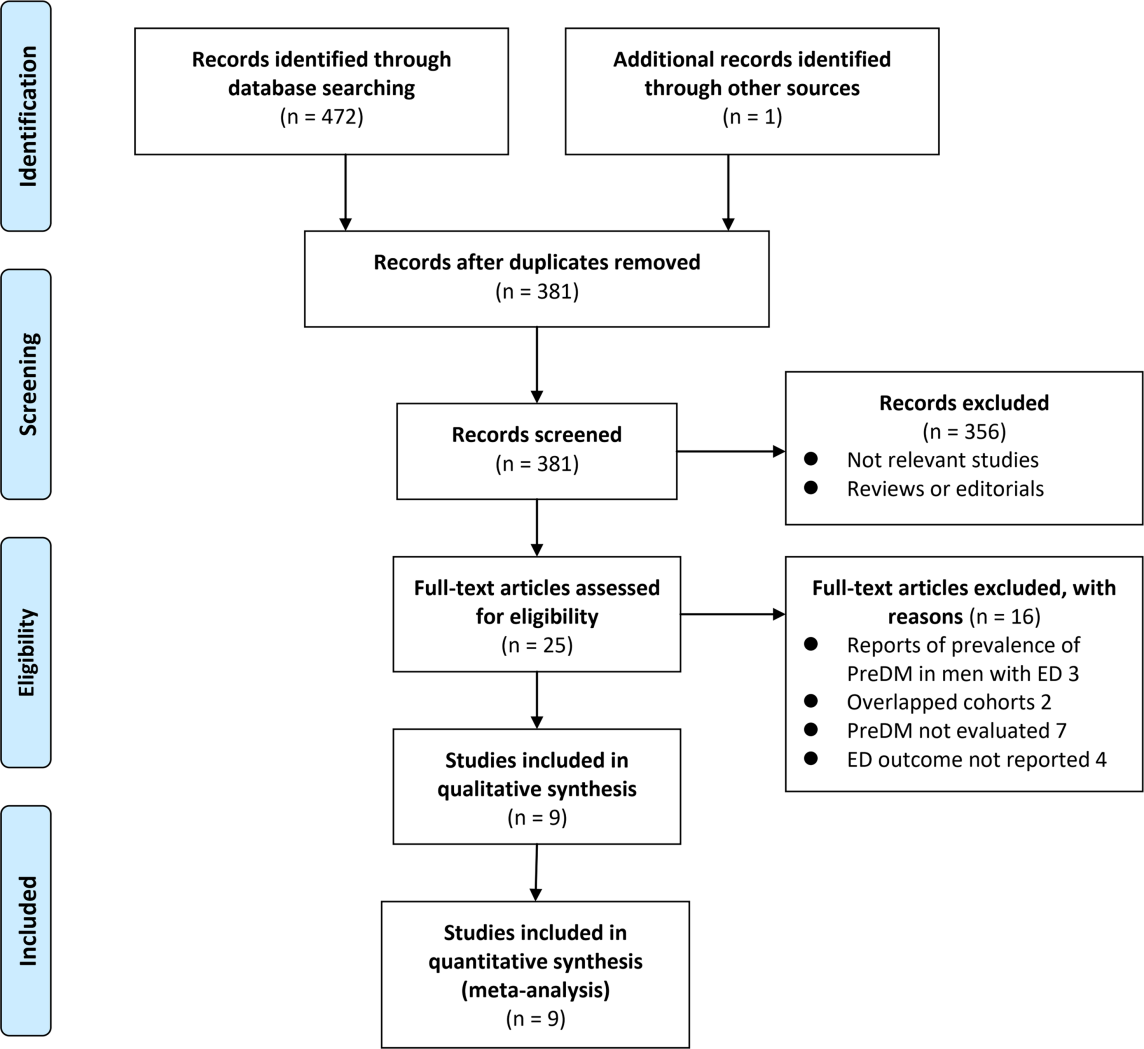
Flowchart of database search and study identification.

The process of database search was summarized in **Figure 1**. Briefly, 381 articles were found *via* initial literature search of the PubMed and Embase databases after excluding of the duplications. Among them, 356 were excluded through screening of the titles and abstracts mainly because they were not relevant to the purpose of the meta-analysis. Subsequently, 25 potential relevant records underwent full-text review. Of these, 16 were further excluded for the reasons listed in **Figure 1**. Finally, eleven observational studies, including five matched case-control studies (14, 18, 19, 21, 22) and four cross-sectional studies (15–17, 20) were included.

### Study Characteristics and Quality Evaluation

The characteristics of the included studies were summarized in **Table 1**. Nine studies with 10980 male adults were included. Eight of them were published in English (14–18, 20–22), and the other one was published in Chinese (19). Three studies included men who attended clinics for sexual dysfunction (14, 16, 20), while the others included men attending the primary care facilities (15, 18, 19) or from the community-derived population (17, 21, 22). The mean ages of the patients varied from 41 to 60 years. Multiple definitions of prediabetes were applied within the included studies, including IGT (14, 17, 19), IFG (15–17, 19), IFG and/or IFG (21, 22), and IGT or IFG or HbA1c (5.7~6.4%) (18, 20). Accordingly, 3862 men had prediabetes. As for the diagnostic methods for ED, the International Index of Erectile Functions (IIEF) questionnaire was applied in seven studies (15, 17–22). For the other two studies, ED was diagnosed based on self-reported symptoms (14) and Structured Interview on Erectile Dysfunction questionnaire (16), respectively. A total of 5511 male adults had ED. Age were controlled in all of the included studies, while in three studies (16, 20, 21), other variables such as body weight, smoking, alcohol drinking, comorbidities, and serum total testosterone were adjusted to a different degree. The NOS scores of the included studies ranged from seven to eight, indicating generally good study quality (**Table 2**).

**Table 1:** Characteristics of the included observational studies.

Study	Country	Design	Participants	Sample size	Mean age (years)	Diagnosis of PreDM	No. of PreDM	Diagnosis of ED	No. of ED	Variables adjusted or matched
Deutsch (14)	USA	Matched CC	Men with suspected sexual dysfunction	183	41.1	IGT	11	Symptom based	51	Age
Grover (15)	Canada	CS	Men 40 years or older attending primary care facilities	3921	56.7	IFG	1552	IIEF questionnaire	1937	Age
Corona (16)	Italy	CS	Men attending outpatient clinic for sexual dysfunction	3451	57.3	IFG	659	SIEDY questionnaire	2240	Age, BMI, smoking, alcohol drinking, and TT levels
Rabijewski (18)	Poland	Matched CC	Men attending the outpatient clinic for glucose metabolism disorders	360	60	IGT or IFG or HbA1c (5.7%~)	176	IIEF questionnaire	97	Age
Ettala (17)	Finland	CS	Community-dwelled men aged between 45~70 years	926	57	IGT or IFG	229	IIEF questionnaire	516	Age
Chen (19)	China	Matched CC	Men attending routine healthcare examination	1500	45.4	IGT or IFG	1000	IIEF questionnaire	386	Age
Rajput (22)	India	Matched CC	Men aged 30~60 years	200	46.6	IGT and/or IFG	100	IIEF questionnaire	137	Age
Krysiak (21)	Poland	Matched CC	Apparently healthy men aged 25~50 years	67	41	IGT and/or IFG	49	IIEF questionnaire	18	Age and body weight
Boeri (20)	Italy	CS	Men attending outpatient clinic for new onset sexual dysfunction	372	54.8	IGT or IFG or HbA1c (5.7%~)	86	IIEF questionnaire	129	Age, BMI, CCI, TT, smoking status, and alcohol consumption

PreDM, prediabetes mellitus; ED, erectile dysfunction; CC, case-control; CS, cross-sectional; IGT, impaired glucose tolerance; IFG, impaired fasting glucose; HbA1c, glycated hemoglobulin; IIEF, International Index of Erectile Functions; SIEDY, Structured Interview on Erectile Dysfunction; BMI, body mass index; CCI, Carlson Comorbidity Index; TT, total testosterone.

**Table 2:** Details of study quality evaluation *via* the Newcastle-Ottawa Scale.

Study	Adequate definition of cases	Representativeness of cases	Selection of controls	Definition of controls	Control for age	Control for other confounders	Exposure ascertainment	Same methods for events ascertainment	Non-response rates	Total
Deutsch (14)	1	0	1	1	1	0	1	1	1	7
Grover (15)	1	1	1	1	1	0	1	1	1	8
Corona (16)	1	0	1	1	1	1	1	1	1	8
Rabijewski (18)	1	0	1	1	1	0	1	1	1	7
Ettala (17)	1	1	1	1	1	0	1	1	1	8
Chen (19)	1	1	1	1	1	0	1	1	1	8
Rajput and Banerjee, (22)	1	0	1	1	1	0	1	1	1	7
Krysiak (21)	1	0	1	1	1	1	1	1	1	8
Boeri (20)	1	0	1	1	1	1	1	1	1	8

### Association Between Prediabetes and ED

**Figure 2:**
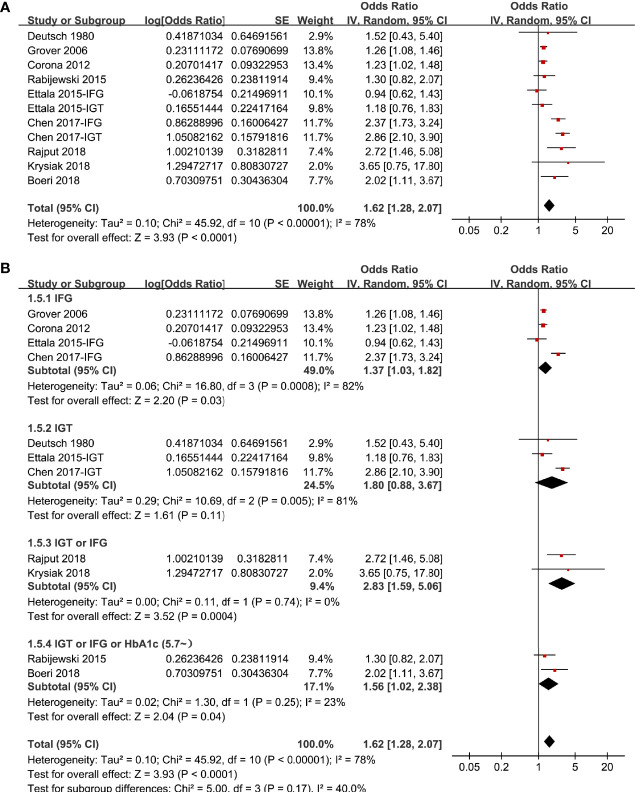
Forest plots for the meta-analysis of the association between prediabetes and ED; **(A)** results of main meta-analysis; and **(B)** results of subgroup analyses according to definition of prediabetes.

**Figure 3:**
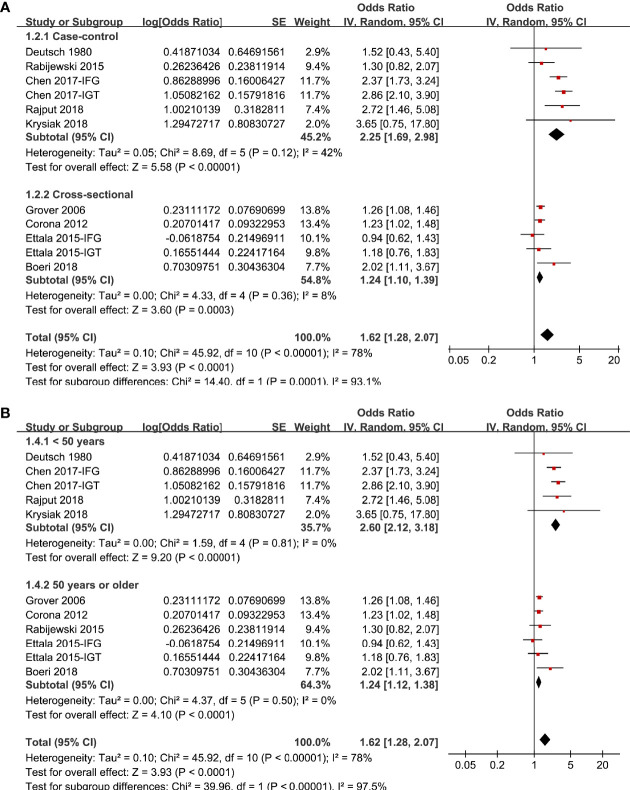
Subgroup analyses the meta-analysis of the association between prediabetes and ED; **(A)** subgroup analyses according to the study design; and **(B)** subgroup analyses according to the mean age of male adults.

**Figure 4:**
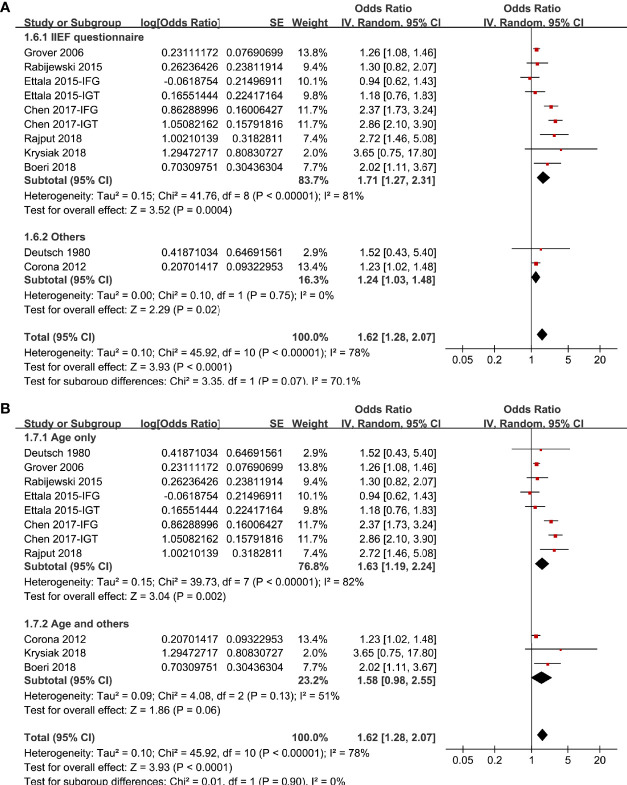
Subgroup analyses the meta-analysis of the association between prediabetes and ED; **(A)** subgroup analyses according to the diagnostic methods for ED; and **(B)** subgroup analyses according to whether additional confounding factors were controlled besides age.

In two studies (17, 19), the association between prediabetes and ED were separately reported for patients with IGT or IFG, and these datasets were separately included into the meta-analysis. Overall, pooling the results of 11 datasets from nine studies (14–22) using a random-effect model showed that compared to those with normoglycemia, men with prediabetes had a higher prevalence of ED (OR: 1.62, 95% CI: 1.28 to 2.07, P < 0.001; **Figure 2A**) with significant heterogeneity (P for Cochrane’s Q test < 0.001, I^2^ = 78%). Sensitive analysis by excluding one study at a time did not significantly change the results (OR: 1.48~1.73, P all < 0.05). In particular, sensitivity analyses excluding the two datasets from the study published in Chinese showed consistent results (OR: 1.32, 95% CI: 1.21 to 1.54, P < 0.001, I^2^ = 34%). Besides, sensitivity analyses excluding the study in which diagnosis of ED was based on self-reported symptoms also showed similar result (OR: 1.63, 95% CI: 1.27 to 2.09, P < 0.001, I^2^ = 80%). Subgroup analyses showed that difference in definitions of prediabetes did not seem to significantly affect the results (P for subgroup difference = 0.17, **Figure 2B**). However, the association between prediabetes and ED seemed to be stronger in case-control studies than that in cross-sectional studies (**Figure 3A**), and in studies with younger men (mean age < 50 years) than in those with older men (mean age ≥ 50 years; **Figure 3B** both P for subgroup difference < 0.05). Particularly, the heterogeneity within subgroups diminished according to the mean ages of the men (I^2^ = 0% in both subgroups; **Figure 3B**), suggesting that mean age of the participants may be a major source of heterogeneity. Additional subgroup analyses showed that the association between prediabetes and ED was not significantly affected by differences in diagnostic tool for ED or controlling of additional variables besides age (**Figures 4A, B**, both P for subgroup difference > 0.05).

### Publication Bias

**Figure 5:**
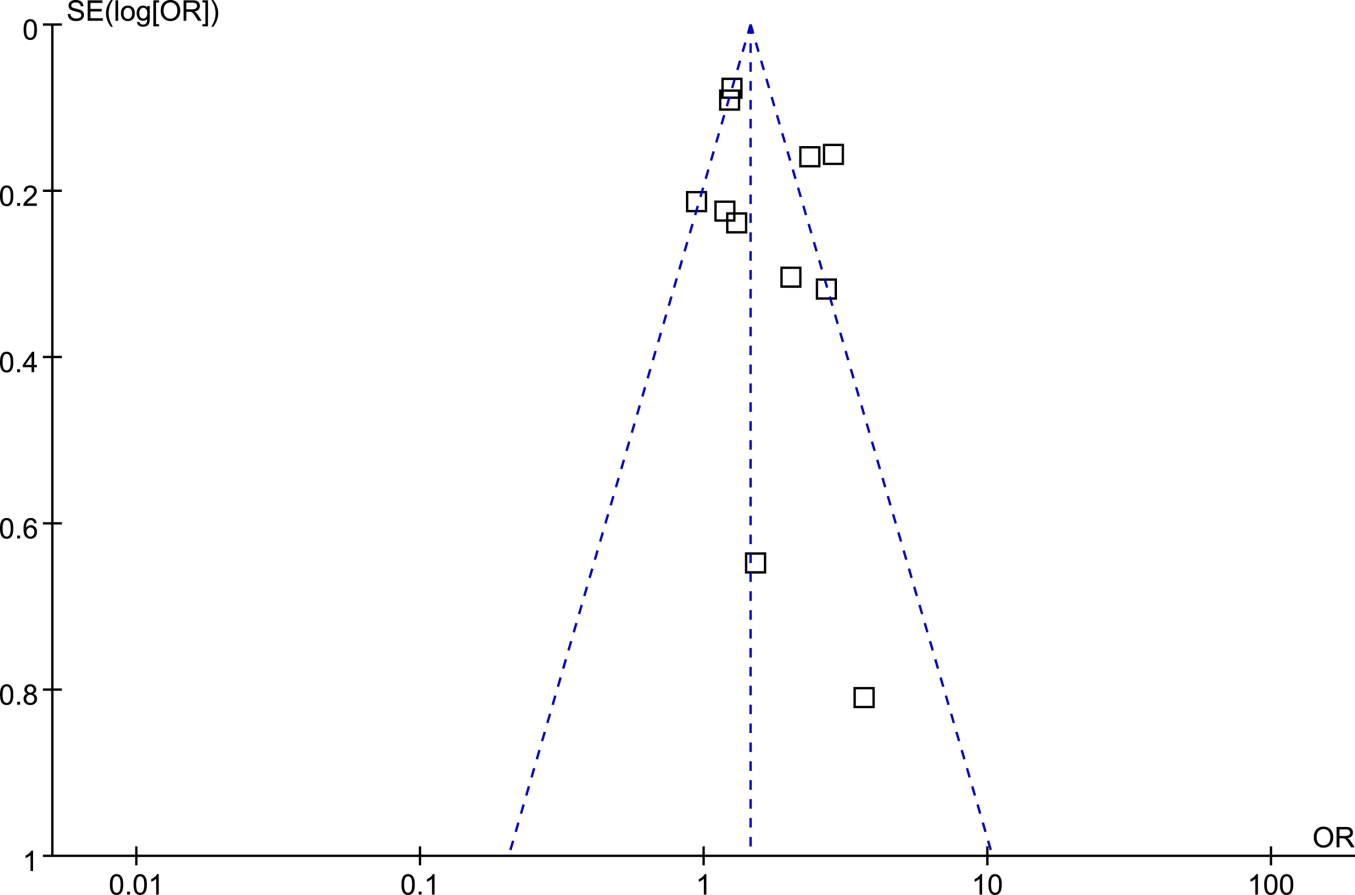
Funnel plots for the publication bias underlying the meta-analysis of the association between prediabetes and ED.

The funnel plots regarding the association between diabetes and ED were shown in **Figure 5**. The funnel plots were symmetrical on visual inspection, suggesting low risk of publication bias. Egger’s regression tests also suggested low risk of publication bias (P = 0.185).

## Discussion

In this meta-analysis, by pooling the results of nine observational studies controlling for age, we found that compared to men with normoglycemia, those with prediabetes were associated with higher prevalence of ED. Further subgroup analysis showed that mean age of the male participants in each study may significantly affect the results, which may substantially contribute to the heterogeneity of the included studies. Specifically, the association between prediabetes and ED seemed to be stronger in studies with younger men (mean age < 50 years) than that in studies with older men (mean age ≥ 50 years). In addition, subgroup analyses showed that difference in definitions of prediabetes, diagnostic methods for ED, and controlling of additional variables besides age did not significantly affect the association. Taken together, these results showed that prediabetes is associated with higher prevalence of ED, which is independent of age and may be stronger in younger men.

To the best of our knowledge, this study is the first meta-analysis which summarized the association between prediabetes and ED. The study has several strengths which should be mentioned for better interpretation of the results. First, since advanced aging has been related to both hyperglycemia (29) and ED (30), it is important to know whether the association between prediabetes and ED could be confounded by aging. Accordingly, only studies matched or adjusted for age was included into the meta-analysis, and the results of the meta-analysis indicated that the association between prediabetes and ED was independent of age of the males. In addition, the robustness of the finding was further evaluated by the “leave-one-out” sensitivity analysis, which showed that the overall result of the meta-analysis was not primarily driven by either of the included studies. Moreover, several predefined subgroup analyses were performed to evaluate the influences of study characteristics on the association. Results showed that the association between prediabetes and ED was consistent and independent of definition of prediabetes, study design, methods for diagnosis of ED, and with or without controlling for variables besides age. It has been confirmed that many factors are involved in the pathogenesis of ED, such as cardiometabolic risk factors besides hyperglycemia (31), central weight gain (32), insulin resistance (33), inflammatory cytokines and leptin (34), and low testosterone (35), all of which may confound the possible relationship between prediabetes and ED. Accordingly, large-scale prospective stuides with adequate adjustment of the above factors are needed to determine the possible independent association between prediabetes and ED.

Interestingly, results of the subgroup analysis showed that the association between prediabetes and ED seemed to be stronger in studies with younger men than that with older men. The potential reasons for the results remain unknown. However, compared to older male participants, younger males were less likely to have various comorbidities that were associated with pathogenesis of ED, such as metabolic syndrome, hypertension, obesity, coronary artery disease, and multiple concurrent medications (36). Accordingly, compared to older male participants who usually have more comorbidity, the adverse influence of prediabetes and hyperglycemia on the pathogenesis and deterioration of ED may be more remarkable in younger men. This is important because ED has traditionally been considered a disease of old age; however, contemporary evidence suggests a growing incidence of ED in men younger (36). Indeed, a previous systematic review of large multinational studies has estimated the prevalence of ED in young men to be as high as 30% (36). Another study showed that age at first presentation for ED significantly decreased over the past decade, which highlighted the importance of careful assessment of ED even at younger age groups (37). Our study expanded these findings by showing that prediabetes in younger men may at risk for ED. From the perspective of prevention, these results highlight the possible importance of screening ED in men with prediabetes, particularly in young male population with prediabetes.

The potential pathophysiological basis for the association between prediabetes and ED may be similar to that between DM and ED. Persistent hyperglycemia is associated with systematic inflammation and endothelial dysfunction, which represents the common denominator leading to vascular ED (38, 39). Besides, hyperglycemia is associated with oxidative stress and autonomic and peripheral neuropathies, both of which were involved in the pathogenesis of ED *via* impairment of sensory impulses from the penis and parasympathetic activity for relaxation of the smooth muscle of the corpus cavernosum (40, 41). Moreover, prediabetes has also been related with reduced testosterone level, which has been recognized as a hormonal risk factor for ED (42). Similarly, prediabetes is shown to be associated with an increased risk of testosterone deficiency, independent of obesity and other cardiometabolic factors (43). Accordingly, screening for low testosterone may be considered in people with prediabetes and symptoms of ED and should be mandatory if ED is diagnosed. Interestingly, a recent study showed that patients with undiagnosed prediabetes were associated with lower rates of response to phosphodiesterase type 5 inhibitors (PDE5i) than normoglycemic men. These findings suggest that even milder forms of glucose impairment are associated with a worse efficacy of PDE5i in men with ED. Future studies are warranted to determine the exact and specific mechanisms underlying the association between prediabetes and ED.

Our study also has limitations. Firstly, all of the included observational studies were cross-sectional, and no longitudinal data was available for the association between prediabetes and incidence of ED. Accordingly, results of the meta-analysis should be validated in large-scale prospective cohort studies. Moreover, the meta-analysis was based on data from study level rather than data of individual patient. Therefore, results of subgroup analysis should be interpreted with caution. In addition, although age was controlled for all of the included studies, we could not exclude the possibility that other factors may confound the association between prediabetes and ED. In this regard, large-scale prospective cohort studies with careful adjustment of possible confounding factors are needed. Finally, a causative relationship between prediabetes and ED should be derived based on the finding of the meta-analysis because this is a meta-analysis of observational studies.

Taken together, results of the meta-analysis showed that prediabetes is associated with higher prevalence of ED, which may be independent of age of the males and may be stronger in young men. Although these findings should be validated in large-scale prospective cohort studies, these results may highlight the possible importance of screening ED in men with prediabetes, particularly in young male population with prediabetes.

## Data Availability Statement

The original contributions presented in the study are included in the article/supplementary material. Further inquiries can be directed to the corresponding author.

## Author Contributions

MJ, SY and CW designed the study. MJ and SY performed literature search, data extraction, and quality evaluation. MJ, BW, and LY performed statistical analyses and interpreted the results. MJ wrote the manuscript. All authors reviewed and revised the manuscript, and approved the manuscript for submission.

## Conflict of Interest

The authors declare that the research was conducted in the absence of any commercial or financial relationships that could be construed as a potential conflict of interest.

## Publisher’s Note

All claims expressed in this article are solely those of the authors and do not necessarily represent those of their affiliated organizations, or those of the publisher, the editors and the reviewers. Any product that may be evaluated in this article, or claim that may be made by its manufacturer, is not guaranteed or endorsed by the publisher.
